# Molecular and Cellular Mechanisms Involved in the Pathophysiology of Retinal Vascular Disease—Interplay Between Inflammation and Oxidative Stress

**DOI:** 10.3390/ijms252111850

**Published:** 2024-11-04

**Authors:** Jovana V. Srejovic, Maja D. Muric, Vladimir Lj. Jakovljevic, Ivan M. Srejovic, Suncica B. Sreckovic, Nenad T. Petrovic, Dusan Z. Todorovic, Sergey B. Bolevich, Tatjana S. Sarenac Vulovic

**Affiliations:** 1 University Clinical Center “Kragujevac”, 34000 Kragujevac, Serbia; srejovic.jovana@gmail.com (J.V.S.); suncica.sreckovic@fmn.kg.ac.rs (S.B.S.); nenad.petrovic@fmn.kg.ac.rs (N.T.P.); dusan.todorovic@fmn.kg.ac.rs (D.Z.T.); 2 Department of Ophthalmology, Faculty of Medical Sciences, University of Kragujevac, 34000 Kragujevac, Serbia; 3 Department of Physiology, Faculty of Medical Sciences, University of Kragujevac, 34000 Kragujevac, Serbia; majanikolickg90@gmail.com (M.D.M.); vladimir.jakovljevic@fmn.kg.ac.rs (V.L.J.); 4 Center of Excellence for the Study of Redox Balance in Cardiovascular and Metabolic Disorders, University of Kragujevac, 34000 Kragujevac, Serbia; 5 Department of Human Pathology, First Moscow State Medical University I.M. Sechenov, Moscow 119435, Russia; bolevich2011@yandex.ru; 6 Department of Pharmacology, First Moscow State Medical University I.M. Sechenov, Moscow 119435, Russia

**Keywords:** retinal vascular diseases, angiogenesis, cellular signaling, vascular endothelial growth factor

## Abstract

Retinal vascular diseases encompass several retinal disorders, including diabetic retinopathy, retinopathy of prematurity, age-related macular degeneration, and retinal vascular occlusion; these disorders are classified as similar groups of disorders due to impaired retinal vascularization. The aim of this review is to address the main signaling pathways involved in the pathogenesis of retinal vascular diseases and to identify crucial molecules and the importance of their interactions. Vascular endothelial growth factor (VEGF) is recognized as a crucial and central molecule in abnormal neovascularization and a key phenomenon in retinal vascular occlusion; thus, anti-VEGF therapy is now the most successful form of treatment for these disorders. Interaction between angiopoietin 2 and the Tie2 receptor results in aberrant Tie2 signaling, resulting in loss of pericytes, neovascularization, and inflammation. Notch signaling and hypoxia-inducible factors in ischemic conditions induce pathological neovascularization and disruption of the blood–retina barrier. An increase in the pro-inflammatory cytokines—TNF-α, IL-1β, and IL-6—and activation of microglia create a persistent inflammatory milieu that promotes breakage of the blood–retinal barrier and neovascularization. Toll-like receptor signaling and nuclear factor-kappa B are important factors in the dysregulation of the immune response in retinal vascular diseases. Increased production of reactive oxygen species and oxidative damage follow inflammation and together create a vicious cycle because each factor amplifies the other. Understanding the complex interplay among various signaling pathways, signaling cascades, and molecules enables the development of new and more successful therapeutic options.

## 1. Introduction

In the adult population, retinal vascular diseases (RVDs), including diabetic retinopathy and retinal vascular occlusions, are the leading causes of visual impairment worldwide. According to the World Health Organization (WHO), an estimated 2.2 billion people worldwide are affected by various forms of visual impairment due to different eye diseases. RVDs represent the majority of retinal diseases caused by various factors, including localized eye diseases and systemic diseases affecting the retinal vasculature [1]. Major ischemic RVDs include diabetic retinopathy, retinopathy of prematurity, age-related macular degeneration, and retinal vascular occlusion. This review summarizes the existing knowledge on the pathophysiology of RVDs, with a focus on inflammation and oxidative stress (OS) as underlying molecular mechanisms.

### 1.1. A Brief Overview of Retinal Physiology and Function

**Figure 1:**
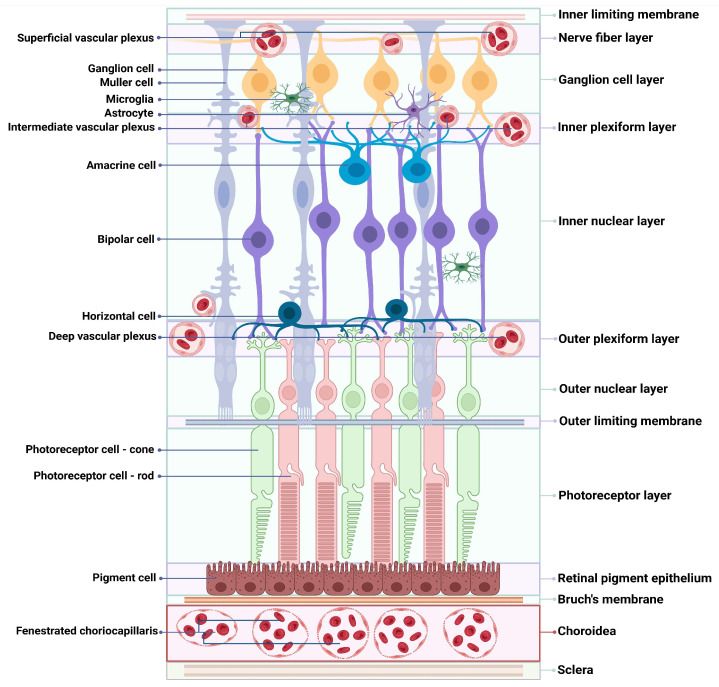
Representation of the physiological structure of the retina and retinal vascularization.

The retina, located in the posterior segment of the eye, is a highly developed nervous tissue consisting of ten different layers [2], six types of neurons (rod, cone, amacrine, bipolar, horizontal, and ganglion), and three types of glial cells (astrocytes, Müller cells, and microglia) (Figure 1) [3,4]. The blood–retinal barrier (BRB) controls the entry of fluid and electrolytes into the extracellular space and maintains homeostasis [5]. The inner component of the BRB is formed by the retinal vascular endothelium (RVE), while the outer BRB consists of the retinal pigment epithelium (RPE), both of which have tight junctions [5]. Anatomically, the RPE serves as a structural barrier separating the retina from the choroid [3]. The morphological structure of the retina, as well as the vascularization of the retina, including the fenestrated capillaries of the choroid located below the retina, are shown in Figure 1.

Light stimulation initiates the transmission of visual signals from photoreceptors to ganglion cells. Ganglion cell axons form the retinal nerve fiber layer (RNFL), which together form the optic nerve. There are two important structures in the central area of the retina: the macula and the optic disk [6]. The fovea, which is located within the macula, contains the highest concentration of photoreceptors and is responsible for central and precise vision [6]. The optic disk, in addition, corresponds to the optic nerve head and serves as the entry point for blood vessels into the eye [7].

Two primary vascular systems that are crucial for maintaining adequate retinal blood flow are retinal and choroidal blood vessels. The retinal vascular system is responsible for 20–30% of blood flow, while the choroidal circulation provides 65–80% of blood flow [8,9]. The vascular network of the inner retina is composed of retinal arterioles and venules that originate from the central retinal artery and drain into the central retinal vein as they cross the inner retinal surface [10], while the outer retina, composed of photoreceptors, remains avascular [11]. The central retinal artery originates from the internal carotid artery via the ophthalmic artery, and the central retinal vein drains directly or indirectly via the superior ophthalmic vein into the cavernous sinus [10]. These retinal vessels run through the center of the optic nerve. The metabolic needs of photoreceptors are primarily met by the choroidal vessels. The choriocapillaris, a dense network of fenestrated capillaries that work closely with the RPE, is located in the choroid [12]. Equipped with specialized tight junctions and transport mechanisms, the RPE facilitates the exchange of nutrients and waste products between the choroidal circulation and the outer retina [12]. The retinal circulation has a high oxygen extraction fraction, which contrasts with the choroidal circulation and is characterized by a low oxygen extraction fraction [10]. In the healthy adult retina, the vasculature is usually mature and remains in a quiescent, nonangiogenic state maintained by a balance between proangiogenic factors, including VEGF, insulin-like growth factor 1 (IGF-1), and erythropoietin (EPO), as well as antiangiogenic factors, such as pigment epithelium-derived factor (PEDF) [13].

### 1.2. Retinal Vascular Diseases—Diabetic Retinopathy

Diabetic retinopathy (DR) is one of the most severe complications of type 1 and type 2 diabetes mellitus (DM) and is the most common RVD leading to visual impairment and blindness [14]. By the year 2030, the global incidence of DR is estimated to reach approximately 454 million people, primarily due to increasing obesity, increased exposure to obesogenic environments, increased life expectancy, and improved diagnostic capabilities for detecting the disease [15]. Factors outside the eye that contribute to the onset and progression of DR include inadequate glycemic control, hypertension, dyslipidemia, prolonged duration of diabetes mellitus, pregnancy, and genetic predisposition [14].

**Figure 2:**
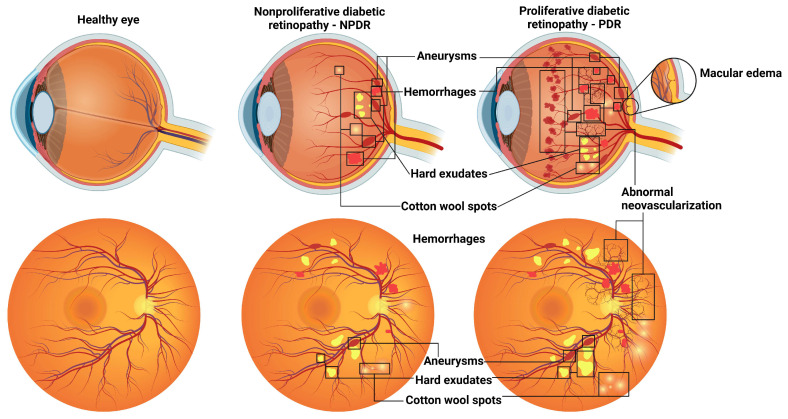
Pathological hallmarks of non-proliferative (NPDR) and proliferative (PDR) stages of DR, including aneurysms, hemorrhages, hard exudates, cotton wool spots in NPDR, and abnormal neovascularization and macular edema, combined with previous for PDR.

One of the characteristic manifestations of DR, first documented by ophthalmoscopy in the 19th century, is the initial appearance of microaneurysms. Over time, these microaneurysms progress to exudative changes, macular edema, ischemia, collateralization, and dilatation of the venules, eventually culminating in proliferative changes [16]. Retinal microvascular changes in DR patients include thickening of the basement membrane; loss of endothelial tight junctions and pericytes; increased vascular permeability; capillary occlusion; microaneurysms; and, in later stages, loss of endothelial cells (ECs) and their transition to mesenchymal cells (Figure 2) [17]. The progressive nature of DR can culminate in retinal ischemia, increased vascular permeability, neovascularization, and macular edema [18]. Ischemia of the retina, manifested as acellular capillaries, is an early sign of DR and has been observed in various experimental models of DR and humans [19]. As a microvascular complication of DM, DR damages not only the retinal microvasculature but also neurons and glial cells [20]. This disease is considered a progressive neurovascular complication characterized by neuronal dysfunction followed by microvascular damage [20].

DR progression typically includes a non-proliferative stage of DR (NPDR) characterized by progressive intraretinal microvascular changes and a proliferative stage (PDR) characterized by the growth of newly formed vessels on the retina or optic nerve head [21]. Common features of NPDR include visible microaneurysms and flame or blot hemorrhages, while PDR is characterized by the growth of new vessels and retinal tissue edema [10]. The most common pathological features of NPDR and PDR are presented in Figure 2. The use of noninvasive imaging techniques, particularly optical coherence tomography (OCT), in conjunction with the introduction of intravitreal anti-vascular endothelial growth factor (anti-VEGF) therapies has significantly improved the early detection and treatment of diabetic macular edema (DME) and led to an improvement in the visual performance of those affected [22]. DME is a specific complication of DR that can occur at all degrees of severity in both NPDR and PDR patients [23]. DME, which occurs in approximately 25% of diabetic patients, is a major factor contributing to visual impairment. It is characterized by central thickening of the fovea, as observed via OCT, and fluorescein leakage, as detected via angiography [16].

DR manifests through a complex interplay among biochemical processes that are particularly influenced by persistent hyperglycemia. These processes include increased levels of advanced glycation end products/receptors (AGE/RAGE), activation of the polyol pathway, activation of protein kinase C (PKC), and activation of the hexosamine pathway, which increases the production of reactive oxygen species (ROS) and, consequently, oxidative damage [24]. Increased OS and inflammatory stimuli result in inflammation, which is a widely found pathogenetic factor in the DR. Increased production of proinflammatory cytokines, including tumor necrosis factor α (TNF-a), interleukin 1 (IL-1), IL-17, and IL-6, contributes to maintaining *circulus vitiosus* and persistent inflammation and OS [24]. Proteomic studies have indicated changes in the plasma levels of several proteins. Retinol-binding protein 1 (RBP1), diphosphoinositol polyphosphohydrolase 3 alpha, and neuroglobin (NGB) were downregulated, while subunit gamma 2 (HBG2) and CD160 antigen18 were upregulated [25]. Vascular endothelial growth factor (VEGF), the production of which is closely related to inflammation and OS, plays a key role in the pathophysiology of DR and DME [24]. An increase in angiogenic platelet-derived growth factor (PDGF) and a decrease in antiangiogenic PEDF in the vitreous fluid of individuals with DR represent common chemokine changes [26].

### 1.3. Retinal Vascular Diseases—Retinopathy of Prematurity

**Figure 3:**
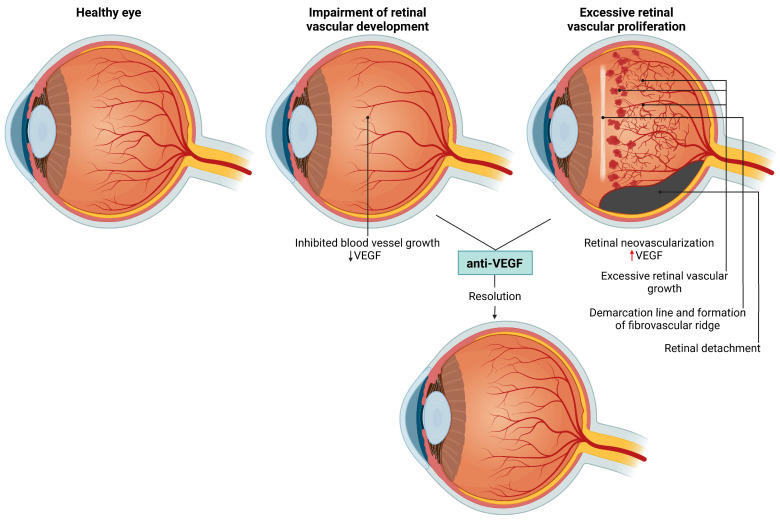
Retinopathy of prematurity (ROP)—different phases of ROP including impaired retinal vascular development in utero induced by low VEGF values, and pathological changes followed by increased VEGF production such as abnormal neovascularization, excessive fibrous tissue growth, and retinal detachment. Anti-VEGF therapy, applied at an early stage as prevention, or when the disease appears, can lead to resolution.

Retinopathy of prematurity (ROP), originally described in 1942 as retrolental fibroplasia, is characterized by abnormal neovascularization of the retina and affects 30% to 50% of newborns with very low birth weight, especially those born preterm [27]. In high-income countries, the incidence of ROP decreases with increasing gestational age at birth, and ROP predominantly, although not exclusively, affects extremely preterm infants (born before the 28th week of gestation) [28]. ROP can lead to severe visual impairment and blindness, while other complications include the development of glaucoma, strabismus, myopia, and amblyopia [29]. The pathophysiology of ROP is multifactorial, with short gestation, low birth weight, and hyperoxia being the most common predisposing factors [30]. The hallmark of ROP pathogenesis is a biphasic pattern characterized by an initial impairment of retinal vascular development and subsequent excessive vascular proliferation that spreads from the retina into the vitreous [28]. Early detection and timely intervention are critical for preventing vision loss in infants with ROP. A key milestone in understanding the pathophysiology of ROP was the discovery of the role of VEGF in the process of retinal neovascularization [31]. Identification of this pathological mechanism plays a crucial role in the development of novel interventions involving intravitreal injections of anti-VEGF antibodies [32]. The anti-VEGF agents ranibizumab, bevacizumab, and aflibercept are frequently used to limit pathological neovascularization of the vitreous in preterm infants [28]. The main pathological features of ROP are presented in Figure 3 and include excessive growth of the retinal blood vessels, formation of the fibrovascular ridge, and, in the final stages, detached retina.

### 1.4. Retinal Vascular Diseases—Age-Related Macular Degeneration

Age-related macular degeneration (AMD) is one of the main causes of blindness due to the deterioration of high-resolution central vision in older people [33]. Numerous modifiable and nonmodifiable risk factors have been associated with the development and progression of AMD [33]. The most common nonmodifiable risk factors include older age (≥65 years), a northern European region, and a family history [34]. AMD affects men and women to a similar extent, although a strong genetic component is also responsible for the occurrence of the disease [33]. The majority of AMD patients (85–90%) suffer from the currently untreatable, atrophic (dry) form of the disease, while the remaining patients (10–15%) develop an exudative (wet) form of AMD [35].

**Figure 4:**
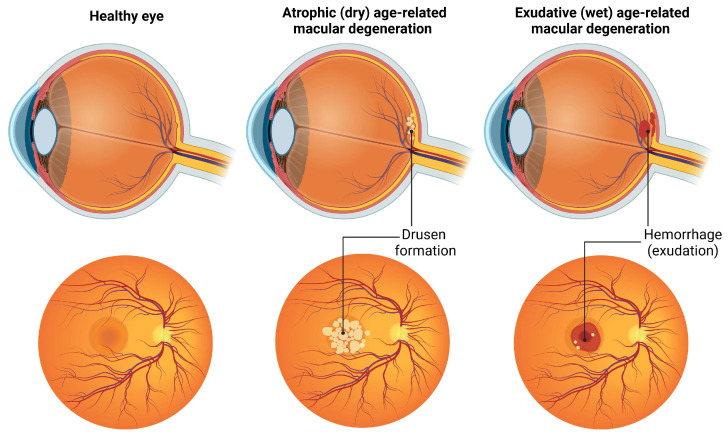
Age-related macular degeneration—the main pathological hallmark of the dry form of AMD is drusen formation, while patients in the wet form of the disease have exudation and hemorrhages as crucial characteristics.

The pathogenesis of AMD is complex and includes genetic susceptibility, age-related disruption of retinal homeostasis, impaired lipid metabolism, immune activation leading to chronic inflammation, OS and ECM dysfunction, all of which contribute to the disease [36]. Pathology changes in patients with AMD affect the inner retinal layers of the macula and adjacent vessels, resulting in impaired central vision [37]. In dry AMD, there is gradual degeneration of the RPE and the outer retinal layers [38]. The wet form is characterized by abnormal choroidal neovascularization that invades both the RPE and the retina in a disorderly manner [38]. In the early clinical stages, both the dry and wet forms of AMD are characterized by the accumulation of lysosomal lipofuscin and extracellular drusen deposits and by degeneration of the RPE [35]. The accumulation of deposits on the retina, known as drusen, is a hallmark clinical feature of AMD and indicates the “dry” form of the disease [37]. Drusen are primarily composed of lipids, proteins, and carbohydrates and can be visualized as small white or yellowish deposits on the macula [33]. Approximately 10–15% of AMD patients develop advanced exudative disease. Differences in the dry and wet form of the AMD are presented in Figure 4.

There are a variety of diagnostic tools for the detection of AMD, including fundus examination, color fundus photography, fundus autofluorescence (FAF), optical coherence tomography (OCT), infrared reflectance (IR), and fluorescein/indocyanine green angiography (FGA) or OCT angiography [38]. The involvement of VEGF in the pathogenesis of neovascular AMD was a breakthrough finding that led to the introduction of anti-VEGF agents as standard treatment for these patients [39]. However, there are still no suitable treatments for the dry form of AMD [35]. Approaches under investigation to slow the progression of AMD include drugs with antioxidant properties, inhibitors targeting the complement cascade, neuroprotective agents, visual cycle inhibitors, gene therapy, and cell-based therapies [39].

### 1.5. Retinal Vascular Occlusions

Acute retinal vascular occlusions, which include both retinal artery occlusions (RAOs) and retinal vein occlusions (RVOs), are the leading cause of visual impairment and loss. These conditions are widely associated with older age and cardiovascular risk factors; however, they differ significantly in pathophysiology and treatment [40]. Appropriate therapeutic strategies are needed to address the distinct features of RAOs and improve visual morbidity and clinical outcomes in these patients.

#### 1.5.1. Retinal Artery Occlusions

The retinal arteries are terminal blood vessels, and occlusion of these vessels leads to complete ischemia of the entire inner retina, which, if blood flow is not restored, is irreversibly damaged for approximately 100 min. Retinal artery occlusion (RAO) is a vascular occlusive disorder that occurs mainly in older people and leads to profound deterioration of vision due to the lack of oxygen available to retinal cells and the optic nerve, resulting in irreversible vision loss [41]. Vascular occlusions in RAOs can be attributed to various factors, such as emboli, blood clots, and lipid plaques, with the most common pathogenic mechanism being a vascular etiology, with stroke and embolism originating from carotid artery plaques [42]. Regarding the site of occlusion, RAOs usually encompass central retinal artery occlusion (CRAO), branch retinal artery occlusion (BRAO), and cilioretinal artery occlusion (CLRAO) [42].

CRAO represents a form of acute ischemic stroke [43] mainly caused by intraluminal thrombosis or embolism and is associated with the presence of various risk factors, such as hypertension, DM and carotid atherosclerotic disease [44]. Literature data show that the occurrence of CRAO is largely associated with ipsilateral carotid artery stenosis, with nearly 40% of patients with CRAO having ipsilateral critical carotid disease [45]. The diagnosis of CRAO relies on the presence of characteristic symptoms such as sudden, painless loss of vision combined with a relative afferent pupillary defect and hypoperfusion of the retina on fundoscopic examination. A prolonged duration of CRAO can lead to a stroke resulting in infarction of the inner retina, including the retinal ganglion cells and their axons that form the optic nerve and central nervous system tissue [46]. Despite extensive research and therapeutic advances in this field, less than 20% of patients with CRAO regain visual function [47].

**Figure 5:**
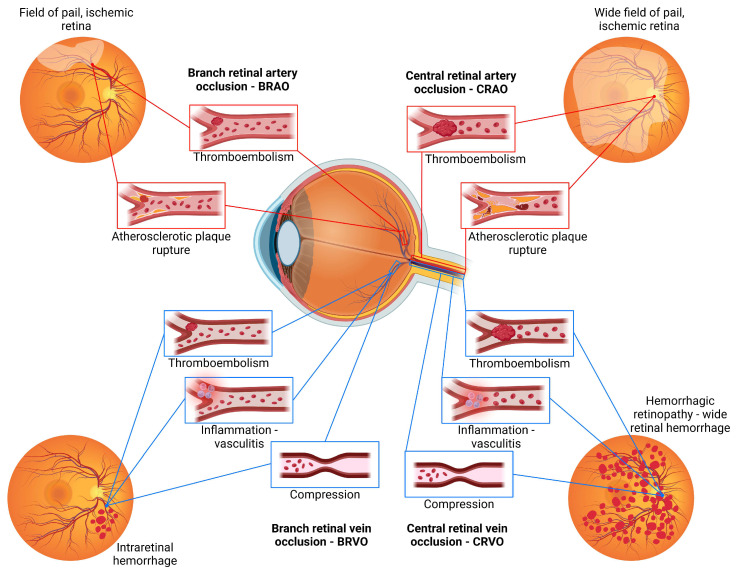
Retinal vascular occlusions—the main etiological factors involved in the pathogenesis of retinal vascular occlusions and clinical presentation of retinal artery and retinal vein occlusion.

BRAO is caused by the occlusion of one or more branches of the central retinal artery and leads to severe visual impairment [48]. The etiology of BRAO is multifactorial and complex, with atherosclerosis of the carotid arteries being the most common underlying disease. Compared to CRAO, BRAO is a relatively rare retinal pathology that requires prompt diagnosis and treatment to improve visual outcomes [48]. The main etiological factors and clinical presentation of retinal artery occlusions are shown in Figure 5.

#### 1.5.2. Retinal Vein Occlusions

After DR, retinal vein occlusions (RVOs) are the second most common type of retinal vascular pathologies and are the main contributors to visual impairment [49]. The most common comorbidities associated with the development of RVOs include hypertension, DM, dyslipidemia, high body mass index, and smoking, while other risk factors include various types of vasculitis, neoplasia, and the use of certain medications [50]. Among the types of RVOs, central retinal vein occlusion (CRVO) and branch retinal vein occlusion (BRVO) are the most common variants, although the latter is more common [51]. In particular, Hemi-CRVO (HRVO), a specific subtype of CRVO, is recognized as a separate entity in certain classification systems [50]. Several studies have shown a correlation between RVO and hyperviscosity due to a high hematocrit, which increases red cell aggregation at low blood flow. In addition, various hematologic disorders, such as thrombophilia and antiphospholipid syndrome, have been associated with an increased risk of RVOs [50].

The exact pathogenesis of RVOs is still not fully understood, but it is postulated that this pathogenesis results from the interaction of three systemic changes, the so-called Virchow’s triad: hemodynamic disturbances leading to venous stasis, degenerative changes in the vessel wall, and an increased tendency toward hypercoagulability of the blood [52]. The impairment of blood flow in one of the retinal veins results from the blockage or constriction that leads to ischemia in the affected retinal region. This blockage can be caused by various factors, such as thrombosis, compression, or inflammation [40]. Retinal ischemia increases vascular permeability, which leads to the accumulation of secondary intraretinal fluid and subretinal fluid in the outer plexiform layer and subretinal space [53]. CRVO results from either a blockage or a significant reduction in venous flow, typically localized at the level of the lamina cribrosa of the optic disc, whereas HRVO involves only one hemisphere of the fundus, with occlusion occurring at either trunk of an abnormally divided intraneural central retinal vein [51].

An important factor in the development of most cases of RVO is arterial disease due to arterial stiffness affecting neighboring veins in the same adventitial sheath within the lamina cribrosa, as in the case of CRVO, or due to venous compression at the site of arteriovenous crossings, degenerative venous changes and hypercoagulability [54]. BRVO is usually caused by an arteriovenous crossing and typically occurs along a large venous branch [51]. The main etiological factors and clinical presentation of retinal vein occlusions are shown in Figure 5.

The multifactorial nature of CRVO may involve local anatomical vulnerability, degenerative changes in vessel walls, and hematological abnormalities [49]. Neovascularization of the retina in the setting of CRVO rarely occurs, but in some cases of ischemic CRVO, angiogenesis reaches the iris and the angle of the eye, resulting in interruption of aqueous humor outflow, increase in intraocular pressure, and “neovascular glaucoma” [55]. CRVO can be divided into two main types: non-ischemic CRVO, also known as venous stasis retinopathy, and ischemic CRVO, often referred to as hemorrhagic retinopathy. Non-ischemic CRVO accounts for approximately 75% of all CRVO cases.

BRVO results from occlusion of any branch of the central retinal vein [56]. In the majority of cases, BRVO is due to arteriovenous crossing, overcrossing of the adjacent artery over the affected vein, or crossover of the affected vein with the adjacent artery [57]. Given that men with BRVO were reported to have a greater chance of having good final vision than women, there is a possible role of sex hormones in the development of BRVO or CRVO [58]. Another possible explanation for these sex differences in visual impairment could be a greater oxygen-carrying capacity in men, which could prevent ischemia and an excessive increase in VEGF [59]. Depending on the anatomical region affected, BRVO can be divided into major and macular BRVO [60]. Major BRVO is an occlusion of a blood vessel draining one of the quadrants, whereas macular BRVO is a vein occlusion within the macula [61]. Neovascularization of the retina is more common in RVOs than in RAOs and can lead to hemorrhage in the case of BRVO [55]. Other complications of BRVO include macular edema, ischemic maculopathy, retinal neovascularization, microaneurysm formation, retinal telangiectasia, and retinal detachment [54]. Macular edema is a leading cause of visual impairment in BRVO patients and, if left untreated, can cause permanent damage to the macular architecture [62]. Treatment of macular edema has been largely revolutionized by targeting VEGF with anti-VEGF agents, indicating the important role of this molecule in the pathophysiology of BRVO [62].

### 1.6. Carotid Artery Stenosis and Ocular Ischemic Syndrome

Carotid artery stenosis is a common risk factor for ocular ischemic complications such as amaurosis fugax, central retinal artery occlusion, and ocular ischemic syndrome, while the ophthalmic artery as a major branch of the internal carotid artery gives rise to the central retinal artery [63]. Reduced blood flow in the carotid artery after stenosis can lead to microcirculatory blockages in both the retina and choroid [64]. Lawrence and colleagues reported that the incidence of ocular symptoms in carotid artery stenosis is greater than 50% [65]. Consequently, without early intervention the metabolism of the retina is disturbed, reducing blood flow to the choroid and causing microstructural changes [66].

**Figure 6:**
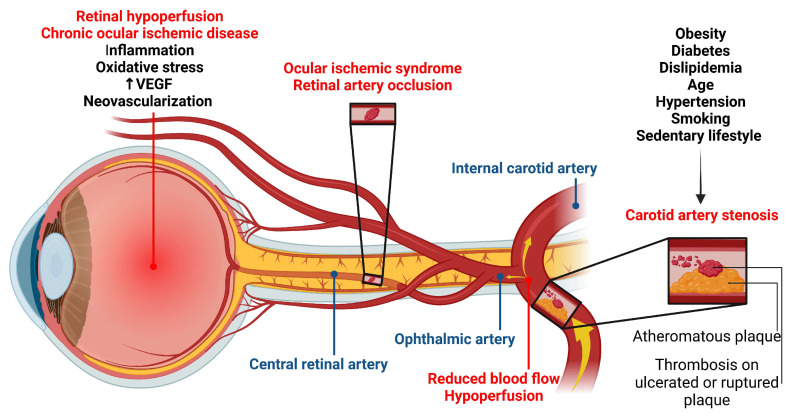
Carotid artery stenosis and ocular implications. Various risk factors contribute to the development of carotid artery stenosis with the consequent development of different aspects of ocular ischemic syndrome and chronic ocular ischemic disease.

Carotid stenosis or carotid occlusion is a significant risk factor for retinal ischemic disease [67]. Lahme and colleagues reported reduced retinal flow density in patients with carotid artery stenosis [68]. Narrowing or blockage of the carotid artery, leading to reduced blood flow in the retina, causes conditions such as ocular ischemic syndrome (OIS) and retinal artery occlusion [67]. The mentioned characteristics are shown in Figure 6. In addition, retinal degeneration is one of the pathologic changes occurring during arterial or venous occlusion or diabetic retinopathy [69]. Chronic ocular ischemic disease can have an insidious onset and a variety of symptoms that, if left untreated, can lead to severe vision loss or serious cerebrovascular events [70]. Several risk factors, including hypertension and carotid occlusion, strongly affect retinal blood flow. Significant improvements have been observed after the use of hypolipidemic medications and carotid surgery [67]. Therefore, early detection of any impairment in ocular blood flow and mild retinal ischemia is essential to prevent ocular ischemic complications.

OIS is characterized by vision loss, orbital pain, visual field changes, and various signs in both the anterior and posterior segments of the eye. Approximately 7.5 cases per million people are diagnosed with OIS each year [71]. This condition is usually undiagnosed, with a poor prognosis, and accompanied by severe ischemic cardiovascular and cerebrovascular diseases. The 5-year survival rate of these patients is 60% [72]. The main cause of OIS is severe carotid stenosis caused by atherosclerosis leading to reduced perfusion pressure of the central retinal artery [71]. This ischemic insult increases the production of vascular endothelial growth factor (VEGF), resulting in neovascularization and increased vascular permeability [73]. OIS is diagnosed in the presence of amaurosis fugax with or without loss of vision, eye pain, conjunctival or episcleral injection, corneal edema, and ocular signs in the anterior and posterior chamber [74]. Fundus fluorescein angiography is commonly used to detect pathological changes, such as non-perfusion of the retina with or without neovascularization and internal carotid artery stenosis [71].

To better understand the pathophysiology of OIS, several experimental models have been developed in rats and mice [75]. In these models, the common or internal carotid artery is surgically occluded or constricted [75] either bilaterally or unilaterally. Researchers commonly perform bilateral occlusions of the common carotid arteries in rats to induce chronic cerebral hypoperfusion, which helps them study neurodegenerative disorders and diseases linked to reduced blood flow [76]. Generally, mice are more prone to ischemic injuries compared to rats, researchers have employed the method of unilateral occlusion of the common carotid artery in mice [75]. These studies have shown that ocular hypoperfusion can lead to similar pathological outcomes, including neovascularization, oxidative damage, and inflammation [75]. Du and colleagues reported increased oxidative and nitrosative stress in an animal model of OIS induced by bilateral common carotid artery, which was related to increased activity of RhoA/MERK1/ERK1/2/iNOS pathways throughout the retina [77].

Early diagnosis and intervention are critical for the treatment of OIS, the resolution of symptoms, and the prevention of permanent vision loss [74]. Treatment options for OIS management are very limited, involving both ocular and systemic treatment [75].

The therapeutic options for described RVDs are shown in Table 1.

## 2. Molecular and Cellular Signaling in the Pathogenesis of Ischemic Retinal Diseases

The retina is one of the most metabolically active tissues in the body and requires a continuous supply of nutrients and oxygen to maintain its function [84]. The RPE plays a critical role in maintaining retinal metabolism by providing essential nutrients and metabolites from the choroidal circulation [85]. The retina and RPE have a symbiotic metabolic relationship in which disruption of RPE homeostasis leads to photoreceptor cell death in the retina and vice versa [85]. Glucose serves as the main fuel source for retinal cells, especially photoreceptors, and supports their high metabolic demands and ATP production through glycolysis and oxidative phosphorylation [86]. Photoreceptors have also been shown to oxidize lipids by fatty acid β-oxidation to generate ATP, which appears to be an important energy substrate for the retina [87].

Ischemia affects retinal energy metabolism, resulting in ATP depletion, accumulation of metabolic byproducts, and dysregulation of metabolic pathways [88]. The extent of retinal tissue damage during ischemia depends on the severity and duration of the blood flow blockage. The region primarily supplied by the blocked vessel forms the infarct core, while the areas supplied by collateral circulation form the ischemic penumbra, where tissue damage is less severe, preserving some function relative to the degree of damage [89]. It was shown that genetically induced pseudohypoxia in RPE cells leads to rapid metabolic stress in these cells and, consequently, to photoreceptor degeneration [90].

The metabolic landscape of the retina is extremely complex due to its cellular composition and functional diversity [91]. Like in the central nervous system, the retina is an immune-privileged tissue that responds only immunologically to a limited extent. The BRB is of particular importance for the maintenance of retinal homeostasis, and it is primarily upheld by tight junctions that restrict the extravasation of leukocytes and intravascular fluid, thus protecting the integrity of the retina [92]. Although the pathogenesis and associated risk factors for major RVDs vary widely, a convergence of common molecular signaling pathways is evident among these conditions. Neovascularization, increased vascular permeability, and inflammation are the characteristic features of different RVDs [93]. The pathogenesis of ischemic retinal diseases involves the activation of deleterious signaling pathways, which include pathways related to energy depletion, excitotoxic injury, dysregulation of calcium homeostasis, OS, and cell death [94]. In the next sections, we will comprehensively describe the main molecular signaling pathways associated with RVDs.

### 2.1. Angiogenesis in Retinal Vascular Diseases

Retinal angiogenesis is a fundamental process involved in both normal vascular development and the pathogenesis of retinal neovascularization in ischemic retinal vasculopathy [95]. The inner BRB is involved in the protection of the inner retina and consists of ECs, the basement membrane, and mural cells [96]. Under physiological conditions, mural cells protect ECs by inhibiting angiogenesis, whereas in retinal diseases such as DR, the loss of mural cells triggers various deleterious processes, including EC death [96]. An imbalance between angiogenic (HIF-1α, PIGF, TGF-beta, FGF, and EDN1) and antiangiogenic (THBS1 and TIMPs) factors is a major factor in the development of retinal neovascularization and can lead to retinal detachment [97]. This phenomenon is common in diseases such as DR, RVOs, and ROP [98].

#### 2.1.1. The Role of Vascular Endothelial Growth Factor

The VEGF family, which includes the molecules VEGF-A, VEGF-B, VEGF-C, VEGF-D, VEGF-E, VEGF-F, and PGF, is formed by alternative splicing of a common starting molecule, resulting in different isoforms [99]. These VEGF isoforms interact with specific VEGF tyrosine kinase transmembrane receptors, of which VEGF receptor 1 (VEGFR-1, also known as Flt-1) and VEGF receptor 2 (VEGFR-2, also known as KDR/Flk-1) are directly associated with angiogenesis [100]. The most potent proangiogenic isoform is VEGF-A (commonly referred to as VEGF), which mediates both retinal and choroidal angiogenesis [101]. Although VEGF maintains the physiological state of the retina [102], excessive production of VEGF, particularly VEGF-A, is involved in the activation of ECs, cell proliferation and migration, and increased vascular permeability.

**Figure 7:**
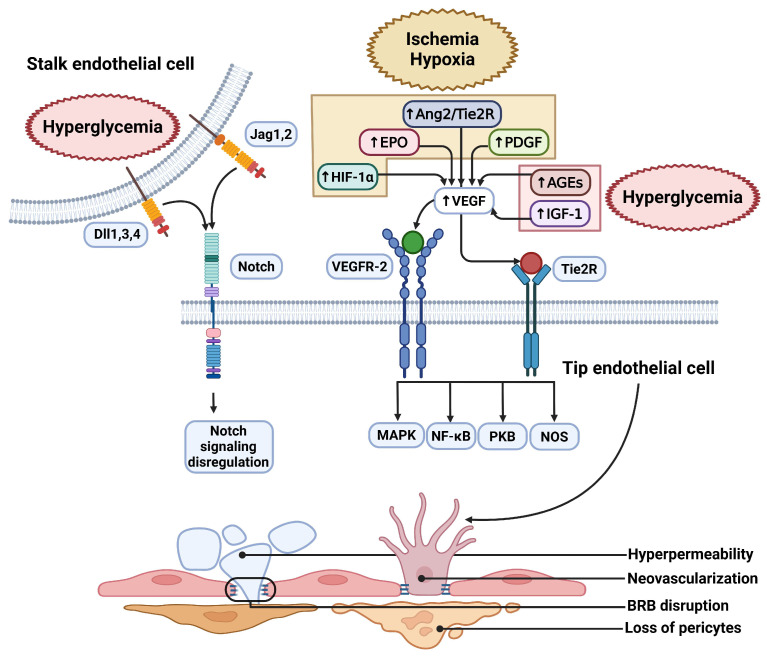
Molecular and cellular signaling in the pathogenesis of ischemic retinal diseases—the key role of VEGF. Abbreviations: Ang2, angiopoietin 2; EPO, erythropoietin; HIF-1α, hypoxia-inducible factor-1α; PDGF, platelet-derived growth factor; AGEs, advanced glycation end products; IGF-1, insulin-like growth factor 1; VEGF, vascular endothelial growth factor; VEGFR2, vascular endothelial growth factor receptor 2; Jag1,2, Jagged 1 and 2; Dll1,3,4, Delta-like ligands 1, 3 and 4; MAPK, mitogen-activated protein kinase; NF-κB, nuclear factor-kappa B; PKB, protein kinase B; NOS, nitric oxide synthase; BRB, blood–retina barrier.

In humans, VEGF, an important determinant of retinal angiogenesis, is produced by RPE cells, astrocytes, Müller cells, and endothelial and ganglion cells [103]. The increase in VEGF production is affected by different factors, such as hypoxia-inducible factor-1α (HIF-1α), EPO, angiopoietin 1 and 2 (Ang1 and Ang2)/Tie2 receptor interaction, platelet-derived growth factor (PDGF), advanced glycation end products (AGEs), and IGF-1 [104,105,106,107,108,109,110]. The relationship between VEGF and other signaling molecules and cascades is shown in Figure 7.

VEGF plays an important role in various retinal diseases, including common retinal diseases such as DR and AMD but also less common diseases such as ROP, sickle cell retinopathy, and retinal vascular occlusion [103]. VEGF is considered an important target for inhibiting retinal neovascularization, and its upregulation contributes to increased vascular permeability, degradation of the extracellular matrix, increased migration and proliferation of vascular ECs, and promotion of angiogenesis [111]. Elevated levels of VEGF have been detected in the vitreous and tear fluids of DR patients and correlate positively with disease severity [112]. In response to ischemic or hypoxic stimuli, the increased production of VEGF induces the phosphorylation of tight junction proteins and increases the permeability of retinal capillaries [113]. In RVO patients, an increased VEGF level is associated with ischemia and is involved in the degradation of the BRB [102]. However, the pathogenesis of ROP is associated with fluctuations in oxygen levels, which also modulate retinal VEGF expression. Hyperoxia leads to the suppression of VEGF, while hypoxia stimulates its expression. This dynamic interplay between oxygen levels and VEGF expression regulates neovascularization. Targeting VEGF through anti-VEGF therapies has revolutionized treatment options for these patients, significantly improving clinical outcomes and alleviating visual impairment. Continued research into VEGF mechanisms may enable further advances in the understanding and treatment of RVDs.

#### 2.1.2. The Angiopoietin-Tie2 Signaling Pathway

Angiogenesis comprises several phases, including vessel sprouting, vessel maturation, and vessel remodeling [107]. Of the four angiopoietins described (Ang1-Ang4), only Ang1 and Ang2 are well characterized with regard to their biological function [114]. Both Ang1 and Ang2 act as peptide ligands that bind to receptor tyrosine kinase with immunoglobulin and epidermal growth factor homology domain 2 (Tie2) receptor. This interaction is essential for the modulation of angiogenesis, vascular stability, and remodeling processes under both normal and pathological conditions [115]. However, Ang1 and Ang2 act in different ways [116]. Ang1 acts as an agonist of the Tie2 receptor and promotes its signaling pathway, thereby promoting vascular survival and inhibiting vascular leakage. Conversely, Ang2 acts as a context-dependent antagonist that inhibits Tie2 signaling in most scenarios but can enhance Tie2 signaling under certain conditions. Ang2 induces vessel wall destabilization through its interaction with integrins or through the inhibition of Ang1 binding to the Tie2 receptor [116] (Figure 7).

The Ang/Tie signaling pathway is critical for the maintenance of vascular stability and is achieved by the delicate balance between the agonistic influence of Ang1 on Tie2 and the antagonistic influence of Ang2 [107]. Five major components of the Tie2 signaling pathway play important roles in ocular angiogenesis: the Ang1 and Ang2 receptors, the Tie1 and Tie2 receptors, and the receptor for vascular endothelial tyrosine phosphatase (VE-PTP) [115]. The Tie2 signaling pathway is specific to vascular ECs [115], but unlike VEGF signaling, it is involved in the maintenance of vascular health by promoting EC survival, maturation, and stability [117] (Figure 7).

In PDR and DMO, Ang2 levels are elevated, suggesting an important role in neovascularization and a possible role in retinal neovascularization and vascular hyperpermeability due to the suppression of Tie2 activity [118]. Preclinical evidence has shown that Ang2 levels increase nearly 30-fold in diabetic rats and are associated with the loss of pericytes [119]. Some authors have suggested that patients with RVDs may benefit more from combined inhibition of Ang-2 and VEGF-A than from anti-VEGF-A monotherapy [107]. This approach may influence the disruption of vascular permeability caused by inflammation, hyperglycemia, or hypoxia [107].

#### 2.1.3. The Notch Signaling Pathway

The Notch signaling pathway involves interactions between Notch receptors (Notch1-4) and their ligands (delta-like ligands [Dll1, Dll3, Dll4] and Jagged [Jag1 and Jag2]) [120]. It is a crucial signaling pathway involved in various aspects of retinal vascular development and function, as well as pathological eye diseases [120]. ECs express multiple components of the Notch signaling pathway both normally involved in physiological processes and under pathological conditions associated with angiogenesis. Notch1, Notch4, Dll1, Dll4, Jag1, and Jag2 are among the major players expressed by ECs [121,122], highlighting their importance in the regulation of vascular development and remodeling.

The involvement of Notch signaling in the modulation of pathological ocular angiogenesis was first established in models of ROP, also known as oxygen-induced retinopathy (OIR) [123]. The Dll4/Notch signaling pathway is involved in the pathogenesis of OIR via two major pathways [123]. In OIR, the inhibition of Dll4/Notch signaling results in increased angiogenic sprouting and regrowth of lost retinal vessels and the suppression of ectopic pathological neovascularization. Inhibition of the Dll4/Notch signaling pathway reduces blood vessel occlusion, resulting in improved blood flow by increasing the expression of vasodilators and decreasing the expression of vasoconstrictors. However, there is evidence that the Notch signaling pathway plays a different role in different microenvironments and Notch inhibition can also exacerbate ischemia-induced angiogenesis [124,125]. In the diabetic retina, hyperglycemia leads to the upregulation of Notch1 ligands such as Dll4 and Jag1 [126] (Figure 7).

Interactions between Notch and VEGF signaling have also been reported [127,128]. VEGF initiates angiogenic sprouting via multiple pathways and simultaneously induces Notch signaling in ECs [120]. During sprouting angiogenesis, at the top of vasodilatation, a specialized type of EC called tip cells exhibit high motility and directionality; in addition, stalk cells provide structural support and proliferate to elongate the developing vessel [129]. Notch activation promotes tip and stalk cell differentiation and suppresses VEGF-induced EC proliferation through p21 [120]. Thus, the Notch signaling pathway has dual functions: it ensures proper EC differentiation and simultaneously acts as a feedback mechanism to regulate VEGF-induced angiogenesis.

#### 2.1.4. The Role of Hypoxia-Inducible Factors (HIFs) in Angiogenesis

Hypoxia-inducible factor-1 (HIF-1) is an important regulator of the hypoxia-genomic response [5]. HIF-1 has two subunits: the oxygen-regulated HIF-1α subunit, whose expression escalates in hypoxic environments; and the continuously active HIF-1β subunit, also known as the aryl hydrocarbon receptor nuclear translocator (Arnt), which is constitutively expressed regardless of oxygen levels [5]. The HIF-1 signaling pathway is known for its central role in cellular responses to hypoxia in various tissues, including the retina [12]. Neovascularization in the retina occurs under pathological conditions that can ultimately lead to retinal blood vessel damage and retinal ischemia. HIF1α is associated with disruption of the BRB and the onset of pathological neovascularization, contributing to visual impairment in ischemic retinal disease [130]. In fact, an ischemic environment triggers increased levels of HIF-1, which in turn stimulates the expression of a group of hypoxia-regulated genes involved in the growth of new vessels in the retina [5]. In addition, HIF-1 activates VEGF, platelet-derived growth factor-B (PDGF-B), placental growth factor (PlGF), and stromal-derived growth factor-1 (SDF-1), all of which are involved in angiogenesis [5]. The levels of HIF-1α and VEGF correlate closely with the severity of DR. Consequently, modulation of VEGF expression represents an important mechanism by which HIF-1α contributes to the progression of DR [131].

### 2.2. Inflammatory Pathways in Retinal Vascular Diseases

Ischemic conditions are followed by an inflammatory response called sterile inflammation [132]. Although both the retina and CNS are considered immune-privileged organs, as neither is capable of eliciting an immunological response [91], recent research has challenged the concept of retinal immune privilege as evidence of contrary events [85]. The role of inflammation in ocular vascular diseases is widely recognized, but its specific cellular origin and underlying signaling pathways are still controversial [133].

Immune privilege is an evolutionary mechanism for protecting components of the immune system; however, once compromised, the entire ocular tissue becomes highly susceptible to damage from inflammation [133]. However, an appropriate inflammatory response requires coordinated interactions between immune cells, endothelial cells, and neurons that initiate signaling cascades mediated by inflammatory factors [127,133].

#### 2.2.1. The Role of Inflammatory Cytokines and Chemokines

Chronic low-grade inflammation has been widely described in both clinical and experimental studies of diabetes [134]. Changes in the levels of various cytokines, chemokines, and adhesion molecules in the ocular tissues of DR patients have been described [24]. Cytokines include various families of molecules with different structures and functions in the immune system. Proinflammatory cytokines such as IL-1 and TNF-α promote inflammation, while anti-inflammatory cytokines such as IL-4 and IL-10 suppress proinflammatory responses [24]. Chemokines are small heparin-binding proteins that mediate the movement of leukocytes to sites of inflammation or injury [24]. In the current literature, four families of chemokines are described: CC chemokines, CXC chemokines, CX3C chemokines, and XC chemokines. In DR, increased levels of chemokines such as MCP-1, CCL2, and CCL5, as well as proinflammatory cytokines such as TNF-α, IL-1β, and IL-6, were observed [24].

The important role of TNF-α has been recognized in DR, with higher levels of this cytokine detected in the serum, vitreous, and aqueous humor of DR patients [135]. In addition, the serum TNF-α concentration is positively correlated with disease severity [136]. TNF-α induces apoptosis, which disrupts the physiological function of the blood vessel wall, resulting in increased retinal vascular permeability and loss of retinal microvascular cells [24]. Elevated levels of the proinflammatory cytokine IL-1β were found in the retinal tissue of diabetic rats [137] as well as in the serum of DR patients [138]. IL-1β has been reported to damage retinal capillary endothelial cells by triggering NF-κB activation and increasing OS. IL-6 is another inflammatory factor associated with increased vascular permeability and angiogenesis induction [139]. Elevated levels of IL-6 have been observed in the aqueous humor and serum of DR patients [140].

#### 2.2.2. The Role of Activated Microglia

**Figure 8:**
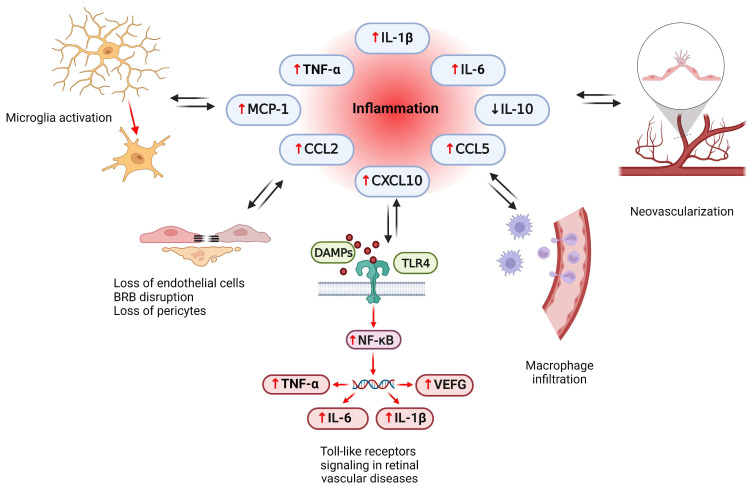
Inflammatory pathways in retinal vascular disorders—the interplay between different interleukins, chemokines, microglia, and development of neovascularization and vascular retinal diseases. Abbreviations: IL-1β, interleukin 1β; IL-6, interleukin 6; IL-10, interleukin 10; TNF-α, tumor necrosis factor α; MCP-1, monocyte chemoattractant protein 1; CCL2, CC motif chemokine ligand 2; CCL5, CC motif chemokine ligand 5; CXCL10, CXC motif chemokine ligand 10; DAMPs, damage-associated molecular pattern molecules; TLR4, toll like receptor 4; NF-κB, nuclear factor-kappa B; BRB, blood–retina barrier.

During retinal development, microglia migrate and are localized in the ganglion cell layer (GCL), the inner plexiform layer (IPL), the outer plexiform layer (OPL), and the nerve fiber layer (NFL) [141]. Retinal microglia have a highly branched structure that maintains retinal homeostasis. However, under pathological conditions, microglia change their phenotype and remain activated to act as neurotoxic mediators during neuroinflammation [4]. Activated microglia migrate to the site of injury where they release proinflammatory factors such as IL-1β, IL-6, and TNF-α and chemokines such as CCL2, CCL4, and CXCL10, leading to disease progression [4]. The roles of different interleukins and chemokines in microglia activation and RVDs are shown in Figure 8.

By producing inflammatory factors, microglia damage the BRB. In a hypoxic environment, microglia are also involved in neovascularization, which leads to secondary lesions and disease progression. During the development of DR, IFN-γ, and IL-6 mediate the activation of microglia via STAT3 phosphorylation, leading to increased secretion of TNF-α, inhibition of kinase activity in the AKT/p70S6 signaling pathway, and loss of pericytes [4]. Excessive IL-6 production activates microglia and augments further overproduction of TNF-α. TNF-α downregulates the expression of zonula occludens-1 and occludin in the RPE and disrupts the tight junctions and the integrity of the outer BRB [142]. In addition, activated microglia increase the permeability of ECs in the inner BRB [142] (Figure 8). The amount of microglia and lipofuscin deposition increases with age in the perivascular and subretinal retina, leading to an increase in AMD risk. Considering that microglia can regenerate indefinitely in the stationary retinal environment [4], targeting microglia in retinal diseases could lead to a significant improvement in the health of patients in this group.

#### 2.2.3. Toll-like Receptor (TLR) Signaling in Retinal Vascular Diseases

Toll-like receptors (TLRs) are a family of type 1 transmembrane receptors that activate innate and adaptive immunity by responding to pathogen-associated molecular patterns (PAMPs) of microorganisms and damage-associated molecular patterns (DAMPs) released by cells during aging or injury [143]. In humans, RPE cells express the genes TLR1-7, TLR9, and TLR10, with TLR1 and TLR3 showing particularly high expression. In addition, the TLR 2-4 and TLR9 proteins are localized in human RPE cells [143].

Hyperglycemic conditions, including DR, lead to increased expression of TLRs, particularly TLR4, in both human and mouse retinal cells, accompanied by increased expression of downstream molecules. This finding suggested that hyperglycemia increases leukocyte activity in the retinal vascular endothelium primarily through TLR4 signaling, although other TLRs are also upregulated in these cells. The pathophysiology of AMD also includes the deposition of extracellular deposits containing DAMPs. This triggers immune responses via TLRs that ultimately reduce the viability of the RPE and photoreceptors through cellular apoptosis. Notably, an association has been observed between genetic polymorphisms and the risk of developing DR and AMD [143]. Retinal ischemia, as in the cases of CRVO and BRVO, releases DAMPs such as HSP70 and HMGB1, which trigger cellular apoptosis via TLR pathways. Elevated TLR levels, particularly TLR4 levels, are associated with retinal ischemia and influence subsequent neovascularization (Figure 8). Given the important role of TLRs in the pathogenesis of noninfectious retinal diseases in particular, the combination of anti-VEGF therapies with TLR inhibition may provide a more durable treatment for these diseases than anti-VEGF therapy alone [143].

#### 2.2.4. Nuclear Factor-Kappa B Signaling Pathway

Activation of nuclear factor-kappa B (NF-κB) promotes leukocyte infiltration, retinal fibrosis, and BRB breakdown through the overexpression of intercellular adhesion molecule-1 (ICAM-1), fibronectin, and CD18 in retinal cells [144]. The role of NF-κB was determined in diabetic mice, where inhibition of this signaling molecule reduced the degradation of cell junction molecules [145]. Furthermore, increased levels of VEGF in diabetic rats stimulate NF-κB activation, which induces angiogenesis [146]. However, suppression of NF-κB reduces retinal neovascularization by decreasing macrophage infiltration and promoting a polarization shift from proinflammatory M1 macrophages to anti-inflammatory M2 macrophages [147]. NF-κB activation plays a central role in the abnormal reactivity of glial cells in diabetic rats [148]. Furthermore, inhibition of NF-κB may be a promising therapeutic approach for treating DR because it reduces inflammation and regulates glial cell activity [148]. Gene set enrichment analysis revealed the involvement of various signaling pathways, such as the IL-6, TNF-α, and TGF-β pathways, in the development of retinal vein occlusion in rabbits [149] (Figure 8). In addition, connectivity map analyses suggest that inhibitors targeting the NF-κB signaling pathway may serve as potential therapeutic options for retinal ischemic diseases [149].

The roles of previously described molecules and signaling pathways are summarized in Table 2.

### 2.3. Oxidative Stress and Vascular Dysfunction

The term OS describes a condition caused by an imbalance between the production of reactive oxygen species (ROS) and the capacity of the antioxidant defense system, resulting in excessive ROS accumulation and tissue damage [150]. Oxidative metabolism occurs mainly in the mitochondria, particularly in the electron transport chain (ETC) and in the endoplasmic reticulum. Even under optimal conditions, electron loss occurs, which contributes to the formation of ROS [150].

Both the anterior and posterior parts of the eye are susceptible to OS, and numerous diseases, including RVDs, are associated with increased levels of free radicals [151]. ROS are generated by the incomplete reduction of molecular oxygen and include hydrogen peroxide (H_2_O_2_), superoxide anion (O_2_^−^), hydroxyl radical (OH^−^), peroxyl radical (ROO^−^), and singlet oxygen (O_2_) [152]. In a physiological state, the cells of the retina are constantly exposed to increased OS caused by strong exposure to light and the function of visual signal transduction pathways, leading to the production of significant amounts of ROS. Factors such as the high oxygen consumption of the outer retina-RPE complex, exposure to strong light, abundance of photosensitizers in both the neurosensory retina and the RPE, and presence of easily oxidizable polyunsaturated fatty acids in the membranes of the outer photoreceptor segment collectively contribute to the initiation of cytotoxic responses [153]. The main sources of ROS in diabetes are oxidative phosphorylation in the mitochondria and the nicotinamide adenine dinucleotide phosphate (NADPH) oxidase system (Nox) [154]. The Nox family includes Nox1-Nox5, dual oxidase 1 (Duox1), and dual oxidase 2 (Duox2), enzymes that can generate either superoxide or hydrogen peroxide, depending on the isoform [155]. Because reactive nitrogen species (RNS) are normally produced in the human body, it is important to mention that physiological concentrations of nitric oxide (NO) are essential for normal vision, while excessive NO concentrations can be harmful [151]. The enzyme responsible for NO synthesis is NOS synthase (NOS), which catalyzes this process in the presence of oxygen and NADPH. There are three isoforms of NOS, neuronal (nNOS), endothelial (eNOS), and inducible (iNOS), which are induced under pathological conditions [156,157].

In addition to other organisms that utilize oxygen, the human body has developed various antioxidant defense mechanisms. These mechanisms include low-molecular-weight and nonenzymatic antioxidants such as vitamins A, C, and E, as well as intracellular antioxidants and antioxidative enzymes, such as catalase, different isoforms of superoxide dismutase (Cu/Zn SOD or SOD1, Mn-SOD, extracellular SOD), reduced glutathione (GSH), and enzymes of the glutathione cycle, such as glutathione peroxidase (GPx) and glutathione reductase (GR) [155].

**Figure 9:**
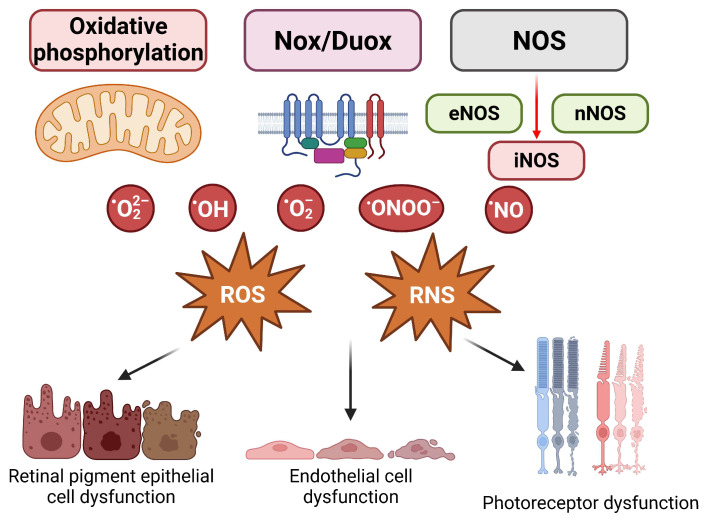
Oxidative stress and damage of retinal cells. Abbreviations: Nox/Duox, nicotinamide adenine dinucleotide phosphate (NADPH) oxidase/dual oxidase; NOS, nitric oxide synthase; eNOS, endothelial nitric oxide synthase; nNOS, neuronal nitric oxide synthase; iNOS, inducible nitric oxide synthase; ^•^HO, hydroxyl radical; ^•^O_2_^2^, peroxide ion radical; ^•^O_2_, superoxide anion radical; ^•^ONOO, peroxynitrite; ^•^NO, nitric oxide; ROS, reactive oxygen species; RNS, reactive nitrogen species.

OS has been identified in several studies as an important factor in the pathophysiology of DR [158]. An increase in O_2_^−^ is most often associated with diabetes and DR and is triggered mainly by hyperglycemia, hypoxia, or proinflammatory cytokines [158]. In addition, reduced levels of antioxidants are also present in the diabetic retina [159]. Müller glial cells play an important role in the antioxidant defense system and the pathogenesis of DR by interacting with cells in all retinal layers [160]. Under hyperglycemic conditions, Müller glial cells exhibit increased expression of heme oxygenase-1 and increased ROS production and consequently respond to OS by producing GSH, which prevents excitotoxicity in the retina [151]. However, decreased GSH levels were observed in the retinas of rats exposed to three weeks of hyperglycemia, which contributes to oxidative damage in DR [161]. An important phenomenon to note is metabolic memory, which represents microvascular changes that persist despite good glycemic control and lead to regression of retinopathy resistance [162]. This imprinting of vascular cells highlights the lasting effects of past periods of poor glycemic control on vascular health [130]. The progression of DR to the PDR stage is particularly associated with OS and reduced antioxidant capacity [163]. In addition, several studies have shown a strong correlation between plasma NO concentrations and the severity of DR. In particular, higher plasma NO concentrations correlate with more severe stages of DR [164]. This relationship suggested that NO plays an important role in the development and progression of DR and may contribute to the vascular dysfunction and retinal damage associated with this disease. The effects of OS on different cell types in the retina are shown in Figure 9.

**Table 2:** Main molecular and/or cellular pathways involved in the pathogenesis of ischemic retinal diseases.

Category	Molecule(s)/Pathway(s)	Key Components	Role in Ischemic Retinal Diseases	Reference
Angiogenesis	VEGF	Particularly VEGF-A	EC activation, Cell proliferation and migration, Increased vascular permeability	[101]
	The angiopoietin-Tie2 signaling pathway	Ang1 (agonist of the Tie2 receptor) Ang2 (context-dependent antagonist that inhibits Tie2 signaling)	Promoting vascular survival, Inhibiting vascular leakage Vessel wall destabilization	[116]
	Notch signaling pathway	Dll4/Notch signaling pathway	Increased angiogenic sprouting and regrowth of lost retinal vessels, Suppression of ectopic pathological neovascularization	[123]
	HIFs	HIF-1α	Disruption of the BRB, Pathological neovascularization	[130]
Inflammatory pathways	Inflammatory cytokines and chemokines	TNF-α, IL-1β and IL-6, Chemokines (MCP-1, CCL2 and CCL5)	Promoting inflammation Promoting apoptosis, Increased retinal vascular permeability and loss of retinal microvascular cells Damage retinal capillary endothelial cells by triggering NF-κB activation Increased oxidative stress Increased vascular permeability and angiogenesis induction Movement of leukocytes to sites of inflammation or injury	[24,139]
	Activated microglia	Migration to the injury site, Release of proinflammatory factors such as IL-1β, IL-6, and TNF-α and chemokines such as CCL2, CCL4, and CXCL10	Disease progression BRB damage neovascularization Loss of pericytes Disruption of the tight junctions and the integrity of the outer BRB Increase in the permeability of ECs in the inner BRB	[4,142]
	Toll-like receptor (TLR) signaling	TLR4 signaling	Responds to PAMPs of microorganisms and damage-associated molecular patterns released by cells during aging or injury Reduces the viability of the RPE and photoreceptors through cellular apoptosis Retinal ischemia Neovascularization	[143]
	Nuclear factor-kappa B signaling pathway	Overexpression of intercellular adhesion molecule-1 (ICAM-1), fibronectin, and CD18 in retinal cells	Promotion of leukocyte infiltration, Retinal fibrosis, and BRB breakdown	[144]
Oxidative stress	Sources of ROS/RNS: Oxidative phosphorylation Nox/Duox NOS	Increased O_2_^−^ Reduced GSH Reduced NO	RPE cell dysfunction Endothelial cell dysfunction Photoreceptor dysfunction	[164]

Abbreviations: VEGF, vascular endothelial growth factor; ECs, endothelial cells; Ang1, angiopoietin 1; Ang2, angiopoietin 2; HIFs, hypoxia-inducible factors; BRB, blood–brain barrier; TNF-α, tumor necrosis factor-α; IL, interleukin; MCP-1, monocyte chemoattractant protein 1; CCL2, CC motif chemokine ligand 2; CCL4, CC motif chemokine ligand 4; CCL5, CC motif chemokine ligand 5; ROS, reactive oxygen species; RNS, reactive nitrogen species; Nox/Duox, nicotinamide adenine dinucleotide phosphate (NADPH) oxidase/dual oxidase; NOS, nitric oxide synthase; O_2_^−^, superoxide anion radical; GSH, glutathione peroxidase; NO, nitric oxide.

**Figure 10:**
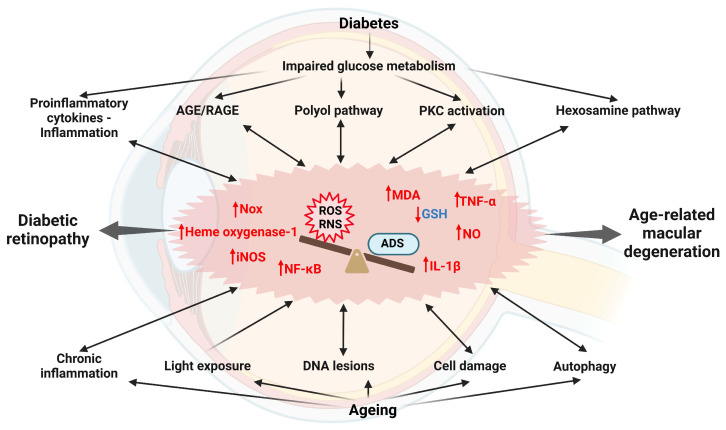
The relation of reactive species and oxidative stress in the development of diabetic retinopathy and age-related macular degeneration. Abbreviations: AGE/RAGE, advanced glycation end products/receptors; GSH, reduced glutathione; IL-1β, interleukin 1β; iNOS, inducible nitric oxide synthase; MDA, malondialdehyde; NF-κB, nuclear factor-kappa B; NO, nitric oxide; Nox, NADPH oxidase system; PKC, protein kinase C; TNF-α, tumor necrosis factor α.

OS is strongly implicated in the development of ROP. Shortly after birth, premature infants experience a rapid shift in oxygen levels as they transition from a low-oxygen environment in utero to a higher oxygen concentration outside the uterus [165]. Both hyperoxia and hypoxia are directly involved in ROP, while an increase in nitro-OS leads to the formation of nitrite, nitrate, and peroxynitrite (ONOO^−^), which contribute to subsequent microvascular damage in the retina [151]. In the pathogenesis of ROP, studies have highlighted the NO_2_^−^-mediated peroxidation process as significant, focusing on nitrosative stress mediators such as trans-arachidonic acid isomers resulting from NO_2_^−^-mediated arachidonic acid isomerization [151]. Specifically, trans-arachidonic acid isomers lead to the upregulation of thrombospondin-1 in retinal ECs, which may further inhibit angiogenesis by inducing the apoptosis of microvascular ECs [166]. In AMD, OS, which occurs mainly in RPE cells, is considered to be one of the main causes of this disease [167] (Figure 9). During periods of high metabolic stress, increased levels of ROS occur in RPE cells, leading to further accumulation of lipofuscin, which contributes to RPE cell dysfunction [151]. Enzymes such as NADPH oxidase and peroxidases are also considered to be additional sources of ROS in AMD [168]. A close link between OS and AMD has been confirmed by elevated levels of OS markers, such as malondialdehyde (MDA) and 8-hydroxy-2′-deoxyguanosine (8-OHdG), in the blood serum of individuals with AMD compared to individuals without AMD [169]. The effects of OS on the development of DR and AMD are shown in Figure 10.

OS is widely recognized as a major factor in the etiology and pathogenesis of various forms of retinopathies. However, despite extensive research, there are still many unresolved questions in understanding the exact mechanisms by which OS contributes to retinopathies [151]. Therefore, ongoing and future studies are crucial for deepening our understanding and unraveling the complexity of OS in the context of RVDs.

## 3. Conclusions

RVDs encompass several entities, the main characteristic of which is a disorder of the eye vasculature; however, these conditions have significant peculiarities related to etiology and pathogenesis. The different signaling cascades involved in the pathogenesis of RVDs create a complex network of interacting molecules that represent potential therapeutic targets. VEGF is one of the crucial mediators involved in the development of different forms of retinal vascular dysfunction; thus, anti-VEGF therapy is currently the most effective form of treatment. The angiopoietin/Tie2 and Notch signaling pathways and HIF-1α are also recognized as important hubs in the pathogenesis of RVDs, especially due to interactions with VEGF. Furthermore, inflammation and OS are key contributors to the development of RVDs. In addition to microglial activation and consequent excitotoxicity, TLR signaling and NF-κB signaling are promising inflammatory targets. Reducing oxidative damage and improving antioxidative defense could be effective adjuvant therapeutic options for all approaches used to treat RVDs. Further experimental and clinical investigations are necessary to elucidate the complex network of interconnected factors involved in the pathogenesis of RVDs.

## Figures and Tables

**Table 1:** The most common RVDs and recommended treatment strategies.

Ischemic Retinal Disease	Recommended Treatments	Reference
DR	Intravitreal anti-VEGF therapy, Corticosteroids, Laser therapy, Vitrectomy, and Conventional drugs for risk factor management	[78]
ROP	Laser therapy, Intravitreal anti-VEGF therapy, β-adrenergic blockers (propranolol)	[79]
AMD	Intravitreal anti-VEGF therapy, Conventional drugs for risk factors, management, and Lifestyle modification	[80]
CRAO	Thrombolysis, Corticosteroids Other conservative treatments (anterior chamber paracentesis, ocular massage, topical intraocular pressure-lowering agents, sublingual isosorbide dinitrate, systemic β-blockade, carbogen therapy, etc.), and Conventional drugs for risk factor management	[81]
BRAO	Thrombolysis (if necessary), Other conservative treatments (ocular massage or paracentesis, hyperbaric oxygenation), and Conventional drugs for risk factor management * Precise therapeutic guidelines are not yet well established.	[82]
CRVO and/or HRVO	Laser therapy, Intravitreal steroids, and Intravitreal anti-VEGF therapy	[51]
BRVO	Laser photocoagulation, Corticosteroids, and Intravitreal anti-VEGF therapy	[51]
OIS	Carotid surgery, Panretinal photocoagulation, Topical steroid and cycloplegics, Topical β adrenergic antagonists or α adrenergic agonists with oral carbonic anhydrase inhibitors, Ciliary body ablation	[51,83]

Abbreviations: VEGF, vascular endothelial growth factor; DR, diabetic retinopathy; ROP, retinopathy of prematurity; AMD, age-related macular degeneration; CRAO, central retinal artery occlusion; BRAO, branch retinal artery occlusion; CRVO, central retinal vein occlusion; HCRVO, hemi-central retinal vein occlusion; BRVO, branch retinal vein occlusion; OIS, ocular ischemic syndrome; * There is no precise therapeutic guidelines for BRAO.
